# Evaluating antibacterial efficiency of orthodontic brackets coated with copper oxide, titanium dioxide and silver nanoparticles 

**DOI:** 10.6026/973206300222147

**Published:** 2026-04-30

**Authors:** Girish Polavaram Vasudeva Setty, Shaik Ziauddhin, Shifin Sainudeen, Anupriya Boopathy, Subash Chandra Nayak, Faraz Hasan, Rubeena Naaz, Md Kafeel Ahmed

**Affiliations:** 1 Private Practitioner, Smile Architect Dental, Bangalore, Karnataka, India; 2 Department of Orthodontic and Dentofacial Orthopedics, Coorg Institute of Dental Science, Virajpet, Karnataka, India; 3 Private Practitioner, Registrar Orthodontist, Aseer Province, Kingdom of Saudi Arabia; 4 Department of Orthodontics and Dentofacial Orthopedics, Sri Venkateshwaraa Dental College, Puducherry, India; 5 Department of Orthodontics and Dentofacial Orthopaedics, Hi -Tech Dental College and Hospital, Bhubaneshwar, Odisha, India; 6 Department of Orthodontics and Dentofacial Orthopaedics, Consultant Orthodontist, Moradabad Uttar Pradesh, India; 7 Private Practitioner, Dental Surgeon, SRK Dental Clinic, Hyderabad, Telangana, India; 8 Department of Periodontology and Implantology, MNR Dental College and Hospital, Sangareddy, Hyderabad, Telangana, India

**Keywords:** Antibacterial, nanoparticles, orthodontic brackets, silver, copper oxide, titanium dioxide, *Streptococcus mutans*

## Abstract

Orthodontic brackets create biofilm niches that promote enamel demineralization and white spot lesions, necessitating effective antibacterial coatings during fixed appliance therapy. Therefore, it is of interest to compare antibacterial activity against *Streptococcus mutans* and *Lactobacillus acidophilus* on 80 brackets coated with CuO-NPs, TiO_2_-NPs or Ag-NPs versus uncoated controls (n=20/group) using sol-gel dip-coating. All nanoparticle coatings significantly outperformed controls (p<0.001); Ag-NPs showed superior reduction (94.2% *S. mutans*, 91.8% *L. acidophilus*), followed by CuO-NPs (87.6%/68.9%) and TiO_2_-NPs (72.4%/68.9%). SEM/EDX confirmed uniform nanoparticle deposition on bracket surfaces. Thus, Ag-NP coating is the optimal strategy to minimize orthodontic biofilm formation and the risk of demineralization, advancing preventive orthodontics through targeted surface modification.

## Background:

Orthodontic appliances are now considered part of modern dental care and many millions of patients worldwide receive bracket-based treatment each year. Nevertheless, the usage of brackets, bands and archwires causes surface irregularities and stagnation sites, which pose a serious threat to oral health care [1]. These device elements have a greater number of bacterial attachment sites and create a safe, secure niche that protects microorganisms against cleaning forces and salivary clearance mechanisms [2]. As a result, patients receiving fixed orthodontic treatment report a meaningful change in their oral microbiome, characterized by increased colonization by cariogenic bacteria. Common etiological agents that contribute to caries in dental treatment and orthodontics include *Streptococcus mutans* and *Lactobacillus* species [3]. These acidogenic and aciduric bacteria are present in the retentive environment formed by orthodontic appliances and generate organic acids during carbohydrate fermentation, which have a demineralizing effect on enamel surfaces [4]. Research has reported that the occurrence of white spot lesions in orthodontic patients is 2 to 96 per cent, with some studies indicating that almost half of all orthodontic patients have at least 1 visible lesion [5]. These are not only the iatrogenic complications that negatively affect aesthetic outcomes, but can also necessitate restorative treatment after the completion of orthodontic treatment. Traditional preventive measures for orthodontic-induced demineralization include improved oral hygiene instruction, fluoride use, antimicrobial mouth rinses and dietary modification [6]. Although these methods have proved to vary in effectiveness, patient compliance is a major limiting factor, especially when the populations of orthodontics are adults, who constitute the majority of orthodontic patients [7]. The emergence of self-cleaning or antimicrobial orthodontic materials that do not require patient participation has thus become a desirable alternative approach to caries prevention during treatment. Nanotechnology has presented novel opportunities to incorporate antibacterial effects into dental materials by using metal and metal oxide nanoparticles [8].

Nanoscale materials exhibit distinct physicochemical characteristics that differ markedly from those of their bulk counterparts, such as increased surface reactivity and antimicrobial activity [9]. The large surface-to-volume ratio of nanoparticles optimizes their affinity for bacterial cells, allowing them to act as antimicrobials at low concentrations. Silver nanoparticles have been widely used in biomedical studies because of their broad-spectrum antimicrobial effects against bacteria, fungi and viruses [10]. Several pathways are involved in the antibacterial action of silver nanoparticles, including challenges to cell membrane integrity, production of reactive oxygen species and disruption of cellular enzymatic activities [11]. Research on the integration of silver nanoparticles into various dental materials has reported substantial reductions in bacterial growth and biofilm development [12]. Another potential antimicrobial agent with photocatalytic capability that generates reactive oxygen species upon exposure to ultraviolet or visible light is titanium dioxide nanoparticles [13]. The photocatalytic performance of TiO_2_-NPs generates hydroxyl radicals and superoxide anions, which oxidize bacterial cell components, causing cell death [14]. Also, titanium dioxide exhibits high biocompatibility and chemical stability; hence, it can be used intraorally [15]. Copper oxide nanoparticles have also become an alternative, as they are cost-effective and exhibit a strong antimicrobial effect due to the release of copper ions and the formation of reactive oxygen species [16]. The antibacterial activities of CuO-NPs are membrane permeabilization, protein denaturation and DNA damage via the oxidative stress pathways [17]. Relativistic research on the use of copper-based antimicrobial agents in dentistry has already demonstrated their potential, although they have not been extensively studied for use on orthodontic brackets [18]. Although there is an increasing focus on orthodontic materials incorporating nanoparticles, few comparative studies have examined various nanoparticle types under standardized conditions [19]. Studies conducted in the past have generally explored individual nanoparticle systems, making it difficult to make direct comparisons across materials because methodologies differ [20]. Therefore, it is of interest to compare the antibacterial activity of copper oxide, titanium dioxide and silver nanoparticles against *Streptococcus mutans* and *Lactobacillus* acidophilus on orthodontic brackets.

## Materials and Methods:

## Study design:

This *in vitro* experimental study was conducted at the Dental Materials Research Laboratory.

## Sample preparation:

Eighty commercially available stainless steel orthodontic brackets (maxillary premolar brackets, 0.022 x 0.028-inch slot, MBT prescription) from a single manufacturer and production batch were used in this study. Brackets were ultrasonically cleaned in acetone for 15 minutes, followed by ethanol for 15 minutes and then rinsed with deionized water. After cleaning, the brackets were dried in a laminar flow hood overnight.

## Group allocation:

Brackets were randomly allocated into four groups of 20 brackets each:

[1] Group 1 (Control): Uncoated stainless steel brackets

[2] Group 2 (CuO-NPs): Brackets coated with copper oxide nanoparticles

[3] Group 3 (TiO_2_-NPs): Brackets coated with titanium dioxide nanoparticles

[4] Group 4 (Ag-NPs): Brackets coated with silver nanoparticles

## Nanoparticle synthesis and characterization:

Commercial nanoparticle powders were obtained from a certified nanomaterials supplier with the following specifications:

[1] Copper oxide nanoparticles: 99% purity, 40-80 nm particle size

[2] Titanium dioxide nanoparticles: 99.5% purity, anatase phase, 20-30 nm particle size

[3] Silver nanoparticles: 99.9% purity, 30-50 nm particle size

Nanoparticle size and morphology were confirmed using transmission electron microscopy (TEM) before coating procedures.

## Coating procedure:

The sol-gel dip-coating technique was employed for nanoparticle deposition. Coating solutions were prepared by dispersing 2% (w/v) nanoparticles in ethanol with 0.5% polyvinylpyrrolidone as a stabilizing agent. Solutions were ultrasonicated for 60 minutes to ensure uniform dispersion.

## The coating protocol consisted of:

[1] Immersion of brackets in the coating solution for 30 seconds

[2] Withdrawal at a controlled rate of 100 mm/minute

[3] Drying at 80°C for 30 minutes

[4] Repetition of the dip-coating cycle three times

[5] Final heat treatment at 200°C for 2 hours to ensure coating adhesion

## Coating characterization:

## Scanning electron microscopy (SEM):

Surface morphology of coated and uncoated brackets was examined using field-emission scanning electron microscopy at an accelerating voltage of 15kV. Images were captured at 5,000x and 20,000x magnifications.

## Energy-Dispersive X-ray Spectroscopy (EDS):

Elemental composition of coated surfaces was analyzed using EDS to confirm the presence and distribution of nanoparticle coatings.

## Coating thickness:

Cross-sectional SEM images were used to measure coating thickness at five randomly selected points per sample.

## Contact angle measurement:

Surface wettability was assessed using a contact angle goniometer with 5 µL deionized water droplets.

## Bacterial strains and culture conditions:

Two reference bacterial strains were used:

[1] *Streptococcus mutans* (ATCC 25175)

[2] *Lactobacillus* acidophilus (ATCC 4356)

Bacterial strains were cultured in Brain Heart Infusion (BHI) broth for *S. mutans* and de Man, Rogosa and Sharpe (MRS) broth for *L. acidophilus*. Cultures were incubated at 37°C under anaerobic conditions (85% N_2_, 10% CO _2_, 5% H_2_) for 24 hours. Bacterial suspensions were adjusted to a 0.5 McFarland standard (approximately 1.5 x 108 CFU/mL) using a spectrophotometer.

## Antibacterial testing:

## Agar diffusion test:

The bracket was placed on Mueller-Hinton agar plates that had been inoculated with standardised bacterial suspensions. Plates were incubated at 37°C for 24 hours with the right atmospheric conditions. Digital calipers were used to measure zones of inhibition four times and the measurements were then averaged.

## Count of colony-forming units (CFU):

Plates with 24 wells (2mL of bacterial suspension) were incubated in the brackets at 37°C overnight. After incubation, the brackets were removed and planktonic bacteria in the suspension were serially diluted (10^-1^ to 10^-6^) and inoculated onto suitable agar media. The number of colonies was determined after 48 hours of incubation and the results were represented as log _10_ CFU/mL.

## Biofilm inhibition assay:

To support biofilm formation, brackets were cultured in a bacterial suspension supplemented with 1% sucrose for 72 hours, with the media replaced after 24 hours. The crystal violet staining technique was used in the quantification of adherent biofilms:

[1] The three times tapping of the brackets with phosphate-buffered saline.

[2] Additionally, staining was done using 0.1 percent of crystal violet over a period of 15 minutes.

[3] Washing to remove the unbound stain.

[4] Staining using 95% ethanol after 10 minutes.

[5] Measurement of absorbance at 595 nm with a microplate reader.

## The percent reduction of bacteria is:

Bacterial counts were also reduced and the percentage was determined with the help of the following formula:

Reduction (percent) = [(CFU control - CFU test)/CFU control) x 100]

## Cytotoxicity assessment:

To measure biocompatibility, human gingiva fibroblasts (HGF) were grown in Dulbecco's Modified Eagle Medium that contained 10% fetal bovine serum. Seeding of cells was performed at 5 x 104 cells/well in 24-well plates with coated brackets. The MTT assay was used to test cell viability after incubation for 24, 48 and 72 hours. The absorbance was measured at 570 nm and cell viability was expressed as a percentage of the control wells without brackets.

## Statistical analysis:

The SPSS software (version 26.0, IBM Corp., Armonk, NY) was used to analyze the data. The test of normality was done by the Shapiro-Wilk test. Means and standard deviations were used as descriptive statistics. Results of the groups were compared using one-way analysis of variance (ANOVA), with which the post-hoc test (Tukey) was conducted to compare the results. Statistical significance was determined at p < 0.05.

## Results:

Scanning electron microscopy revealed successful nanoparticle deposition on bracket surfaces across all three experimental groups. Uncoated control brackets displayed the typical smooth metallic surface of stainless steel with minor manufacturing scratches. Nanoparticle- coated brackets showed uniform surface coverage with characteristic particle morphologies visible at high magnification. Silver nanoparticle coatings appeared as spherical particles distributed evenly across the surface. Copper oxide and titanium dioxide coatings displayed more clustered arrangements with some particle aggregation. Energy-dispersive X-ray spectroscopy confirmed the elemental composition of each coating type. The EDS spectra for Ag-NPs-coated brackets showed prominent silver peaks, CuO-NPs-coated brackets demonstrated copper and oxygen peaks and TiO_2_-NPs-coated brackets revealed titanium and oxygen peaks. Control brackets showed only iron, chromium and nickel peaks characteristic of stainless steel. The mean coating thickness was 1.82 ± 0.34µm for CuO-NPs, 1.67 ± 0.28 µm for TiO_2_-NPs and 1.74 ± 0.31 µm for Ag-NPs, with no significant difference among groups (p=0.412). Contact angle measurements showed that all nanoparticle coatings increased surface hydrophilicity compared to uncoated controls (control: 78.4 ± 4.2°C; CuO-NPs: 62.3 ± 3.8°C; TiO_2_-NPs: 58.7 ± 4.1°C; Ag-NPs: 65.2 ± 3.5°C) (Table 1 - see PDF). All nanoparticle-coated brackets demonstrated significantly greater antibacterial activity against *S. mutans* compared to uncoated controls. The agar diffusion test revealed measurable zones of inhibition for all coated groups, whereas control brackets showed no inhibition zones. Silver nanoparticle-coated brackets produced the largest zones of inhibition (8.42 ± 1.23 mm), followed by CuO-NPs (6.87 ± 1.08 mm) and TiO_2_-NPs (4.56 ± 0.89 mm). Colony-forming unit counts confirmed the antibacterial efficacy of nanoparticle coatings. The mean log10 CFU/mL for *S. mutans* was 8.24 ± 0.18 in the control group, 6.31 ± 0.24 in the TiO_2_-NPs group, 5.72 ± 0.21 in the CuO-NPs group and 4.86 ± 0.19 in the Ag-NPs group. The percentage reduction in bacterial counts compared to control was 72.4% for TiO_2_-NPs, 87.6% for CuO-NPs and 94.2% for Ag-NPs. Similar trends were observed for *L. acidophilus*, with all nanoparticle coatings demonstrating significant antibacterial effects. Zones of inhibition measured 7.68 ± 1.15 mm for Ag-NPs, 6.24 ± 0.97 mm for CuO-NPs and 4.12 ± 0.82 mm for TiO_2_-NPs. The mean log_10_ CFU/mL values were 8.12 ± 0.21 for control, 6.48 ± 0.26 for TiO_2_-NPs, 5.91 ± 0.23 for CuO-NPs and 5.02 ± 0.20 for Ag-NPs. Bacterial reduction percentages were 68.9% for TiO_2_-NPs, 84.3% for CuO-NPs and 91.8% for Ag-NPs (Table 2 - see PDF). Crystal violet staining demonstrated significant biofilm inhibition by all nanoparticle coatings. For *S. mutans* biofilms, absorbance values (OD595) were 1.84 ± 0.22 for control, 0.68 ± 0.14 for CuO-NPs, 0.92 ± 0.18 for TiO_2_-NPs and 0.42 ± 0.11 for Ag-NPs. The corresponding biofilm inhibition percentages were 63.0% for CuO-NPs, 50.0% for TiO_2_-NPs and 77.2% for Ag-NPs. For *L. acidophilus* biofilms, absorbance values were 1.72 ± 0.19 for control, 0.74 ± 0.16 for CuO-NPs, 0.98 ± 0.17 for TiO_2_-NPs and 0.48 ± 0.12 for Ag-NPs. Biofilm inhibition percentages were 57.0% for CuO-NPs, 43.0% for TiO_2_-NPs and 72.1% for Ag-NPs. Cell viability assessment using human gingival fibroblasts demonstrated acceptable biocompatibility for all nanoparticle coatings over the 72-hour evaluation period. At 24 hours, cell viability relative to control was 94.2 ± 4.1% for CuO-NPs, 97.8 ± 3.2% for TiO_2_-NPs and 95.6 ± 3.8% for Ag-NPs. At 72 hours, viability remained above 85% for all groups, with TiO_2_-NPs showing the highest viability (93.4 ± 4.6%), followed by Ag-NPs (89.2 ± 4.8%) and CuO-NPs (86.7 ± 5.2%) (Table 3 - see PDF).

## Discussion:

This research paper will show that nanoparticle coatings can enhance the ability of stainless steel orthodontic brackets to act as antibacterial agents, with silver nanoparticles showing the strongest effect, followed by copper oxide and titanium dioxide nanoparticles. These results confirm the rejection of the null hypothesis and provide useful comparative data for selecting the antimicrobial coating to use on orthodontic implants. The excellent antimicrobial effect of the silver nanoparticle coating in this experiment is consistent with the well-documented antimicrobial activity of the noble metal silver [21]. The pathways of silver's antibacterial effect include direct membrane damage from attachment and penetration, the formation of reactive oxygen species that cause oxidative stress and the disruption of DNA replication and protein synthesis [22]. The 94.2% decrease in *S. mutans* counts using Ag-NPs-coated AgNPs is a clinically significant reduction in bacterial suppression that may be effective in reducing the risk of demineralization during an orthodontic procedure. The antibacterial efficacy results (Ag-NPs higher than CuO-NPs, higher than TiO_2_-NPs) also correlate with comparative studies in other dental applications [23]. This ranking is likely due to the varied primary antibacterial actions of different nanoparticle types. Silver nanoparticles exhibit direct antimicrobial activity due to ion release, membrane interactions and a sustained antibacterial effect without external activation [24]. By contrast, the titanium dioxide photocatalytic mechanism is light-dependent and might not have been ideal under the conditions of incubation adopted [25]. The copper oxide nanoparticles exhibited a moderate antibacterial effect, reducing the incidence of *S. mutans* by 87.6%. The antimicrobial effect of CuO-NPs is caused by the release of copper ions, which disrupt bacterial membrane integrity and generate reactive oxygen species via Fenton-like reactions [26]. Past studies have established that copper-based antimicrobial surfaces can reduce bacterial colonization in healthcare facilities and therefore, can be used in dental practice [27]. A recent *in vitro* study by Ameli *et al.* found that SS brackets coated with CuO, TiO_2_ and HA-silver nanoparticles all showed comparable antibacterial effects against *Streptococcus mutans* over 90 days, with CuO showing slightly better short-term inhibition. This suggests that nanoparticle coatings may help reduce bracket-associated cariogenic biofilm formation, potentially lowering the risk of white spot lesions and caries during orthodontic treatment [28]. The relative cheapness of copper over silver can make CuO-NPs coating an attractive option when antibacterial potency is not required to the maximum. The biofilm inhibition outcomes showed that biofilm formation by all nanoparticle coatings was significantly reduced, with Ag-NPs exhibiting a 77.2% reduction in *S. mutans* biofilm formation. The prevention of biofilms is especially relevant in orthodontic practice because mature biofilms exhibit much greater resistance to antimicrobial agents than planktonic cells do [29]. The capacity of nanoparticle coatings to prevent initial bacterial adhesion and biofilm formation may offer sustained demineralization protection over the duration of the treatment. The clinical significance of the observed antibacterial activity against *S. mutans* and *L. acidophilus* is that these two species are the main cariogenic bacteria in orthodontic demineralization [30]. It has been shown that the levels of both bacterial types are elevated in orthodontic patients and that colonization preferentially occurs in the spaces sheltered by brackets and arch wires [31]. The antimicrobial coating will target the pathogens responsible for orthodontic- related white spot lesions. Surface characterization has shown that the use of nanoparticles via surface modification on bracket surfaces altered surface roughness and wettability. The higher surface roughness associated with nanoparticle deposition may, in theory, enhance bacterial adhesion. Still, the antibacterial action of the surfaces was clearly negative, counteracting the adverse effects of the surface topography change [32]. Lower contact angles, which suggest greater hydrophilicity, can also improve oral care by facilitating salivary wetting and debris removal from bracket surfaces. The cytotoxicity findings indicated that all nanoparticle coatings exhibited acceptable biocompatibility, with cell viability above 85 percent in all experimental groups at 72 hours. These results are comparable to earlier research showing that adequately designed nanoparticle coatings can achieve antibacterial effects without inducing c ytotoxicity in human cells [33]. Titanium dioxide exhibited the greatest biocompatibility, which is consistent with its broad use in biomedical applications and its known safety profile [34]. The slightly lower viability observed with CuO-NPs could be attributed to cytotoxicity mediated by copper ions. Still, the amounts were within the acceptable limits. Nanoparticle coatings were produced using the sol-gel dip-coating technique in this study and uniform coatings with similar thickness were observed across all experimental groups. The benefits of this coating technique are that it is simple and cost-effective as well as scalable to an industrial scale [35]. The adhesion of the coating after heat treatment seemed sufficient under in vitro testing conditions, but its long-term performance in clinical settings, such as mechanical loading and chemical exposure, needs further study [36]. Clinical implications of such findings are that they may be used to develop self-disinfecting orthodontic brackets to minimize demineralization without relying on patient compliance. Research has consistently shown a lack of oral hygiene compliance as a primary risk factor for the development of white spot lesions, especially among adolescent patients [37]. Including the protective measure of coating nanoparticles onto commercially available brackets may offer a supplementary procedure for protection that is not dependent on the patient's actions. There are several limitations of this study worth noting. Although this in vitro experimental design provides control over the comparative efficacy of antibacterial agents, it does not fully model the complex oral environment, which contains salivary proteins, dietary components and polymicrobial communities. The 72-hour biofilm testing time might not reflect the long-term biofilm dynamics during extended orthodontic treatment. Also, the stability of nanoparticle coatings under simulated clinical conditions such as brushing, mastication and exposure to acidic drinks needs to be evaluated [38]. Future studies are needed to examine the stability of nanoparticle surfaces over long-term clinical use, determine the efficacy of antibacterial agents against multispecies biofilms and conduct *in vivo* studies to assess how nanoparticles affect the incidence of demineralization in clinical literature. The optimization of nanoparticle concentration and coating parameters to achieve the maximum antibacterial effect while keeping potential negative effects at a minimum permits further research.

## Conclusion:

Nanoparticle coatings markedly enhance orthodontic brackets' antibacterial activity against *S. mutans* (94.2%) and *L. acidophilus* (91.8%), with Ag-NPs superior, followed by CuO-NPs and TiO_2_-NPs. These coatings effectively inhibit biofilm while maintaining biocompatibility, offering a compliance-independent strategy to prevent demineralization and white spot lesions. Thus, Ag-NPs provide optimal performance and CuO-NPs are a cost-effective alternative. However, clinical trials are needed to validate long-term *in vivo* efficacy.
